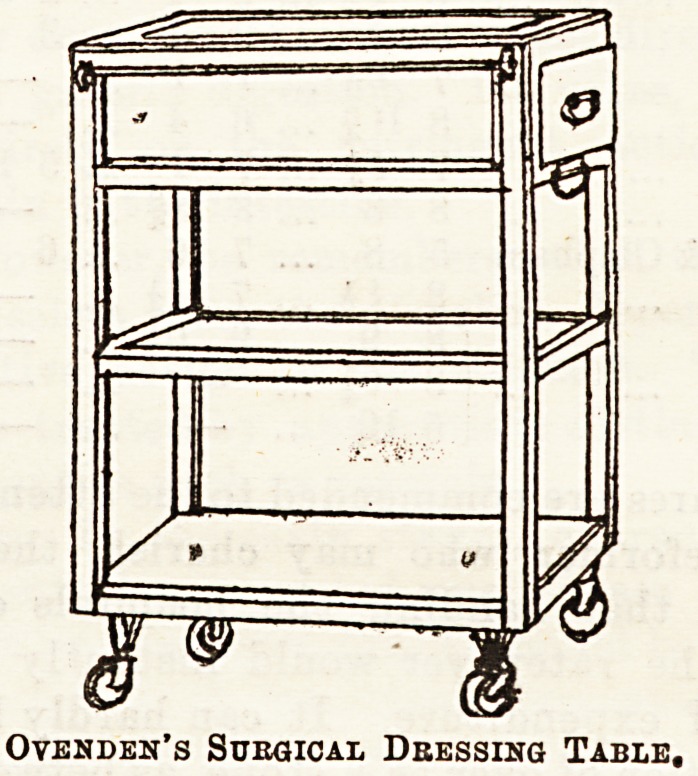# Practical Departments

**Published:** 1896-06-27

**Authors:** 


### PRACTICAL DEPARTMENTS.

### PATENT INTERCHANGEABLE RUBBER STAIR TREADS.

The rubber stair treads manufactured by Mr. W. Gooding, North Road, Holloway, N., appear to possess excellent qualifications from several points of view for the stairs of institutions and public buildings where there is much tramping up and down. The treads consist of an iron "keeper" or frame, pierced with a number of square holes, through which rubber blocks are placed, the blocks forming the wearing service and giving a firm foothold under all circumstances. One advantage is naturally that the rubber blocks may be renewed as often as required at comparatively small cost, and that when those in the centre, where the wear is hardest, become at all worn they may be transferred to the end holes. The treads are very safe in wet and slippery weather, when ordinary iron steps are apt to be dangerous; and are also less noisy. The idea is a good one, and the treads are said to have given every satisfaction where they have been in use for some years. They have just been adopted by the London County Council for fire engine drivers' foot-boards, etc. Mr. Gooding is to be congratulated on a really practical invention.

### SURGICAL DRESSING TABLE.

"Ovenden's Surgical Dressing Table," of which we give an illustration by permission of the makers, is excellent in every way for its purpose. Made in polished wood with glass shelves, it is very light, and moves easily on small rubber wheels. The top drawer is a sliding one, pulling through from one side to the other, so as to be equally available from either end, and the table generally is just the right size to wheel from bed to bed with all the needful surgical paraphernalia.

Ovenden's Surgical Dressing Table.

The table can be ordered from Mr. H. Ovenden, of Tunbridge Wells (whose name, by the way, is also known in connection with an admirable "locker"), through Messrs. Elt and Co., 43, Southampton Buildings, Holborn.

### THE "BUOYANT" BED-REST.

This bed-rest is on the same principle as one already noticed in these columns, the only difference being in the manner of application. It is of the hammock nature, consisting simply of a piece of canvas attached to the head and foot rail of the bed by straps, fastened to wooden rollers. By means of a lever arrangement the head and shoulders of a patient can be raised slowly from a horizontal to a sitting position. A support of this kind is far more comfortable to an invalid in many cases than the hard frame of a wooden or iron bed-rest, and can be easily washed and disinfected when necessary. The inventors and manufacturers are Messrs. Oldfield and Studdard, 67, New Street, Huddersfield.

## Figures and Tables

**Figure f1:**